# Evolutionary History of Contagious Bovine Pleuropneumonia Using Next Generation Sequencing of *Mycoplasma mycoides* Subsp. *mycoides* “Small Colony”

**DOI:** 10.1371/journal.pone.0046821

**Published:** 2012-10-08

**Authors:** Virginie Dupuy, Lucía Manso-Silván, Valérie Barbe, Patricia Thebault, Emilie Dordet-Frisoni, Christine Citti, François Poumarat, Alain Blanchard, Marc Breton, Pascal Sirand-Pugnet, François Thiaucourt

**Affiliations:** 1 Centre International de Recherche en Agronomie pour le Développement, UMR CMAEE, Montpellier, France; 2 CEA/DSV/IG/Genoscope, Evry, France; 3 Université de Bordeaux, Centre de bioinformatique et de génomique fonctionnelle, CBiB, Bordeaux, France; 4 Institut National de Recherche Agronomique, école vétérinaire de Toulouse UMR 1225, Toulouse, France; 5 Université de Toulouse, INP, ENVT, UMR1225, IHAP, Toulouse, France; 6 Agence Nationale de Sécurité Sanitaire, Laboratoire de Lyon, UMR mycoplasmoses des ruminants, Lyon, France; 7 Université de Bordeaux, UMR1332, Villenave d'Ornon, France; 8 Institut National de Recherche Agronomique, UMR1332, Villenave d'Ornon, France; Institut de Pharmacologie et de Biologie Structurale, France

## Abstract

*Mycoplasma mycoides* subsp. *mycoides* “Small Colony” (MmmSC) is responsible for contagious bovine pleuropneumonia (CBPP) in *bovidae*, a notifiable disease to the World Organization for Animal Health (OIE). Although its origin is not documented, the disease was known in Europe in 1773. It reached nearly world-wide distribution in the 19^th^ century through the cattle trade and was eradicated from most continents by stamping-out policies. During the 20^th^ century it persisted in Africa, and it reappeared sporadically in Southern Europe. Yet, classical epidemiology studies failed to explain the re-occurrence of the disease in Europe in the 1990s. The objectives of this study were to obtain a precise phylogeny of this pathogen, reconstruct its evolutionary history, estimate the date of its emergence, and determine the origin of the most recent European outbreaks. A large-scale genomic approach based on next-generation sequencing technologies was applied to construct a robust phylogeny of this extremely monomorphic pathogen by using 20 representative strains of various geographical origins. Sixty two polymorphic genes of the MmmSC core genome were selected, representing 83601 bp in total and resulting in 139 SNPs within the 20 strains. A robust phylogeny was obtained that identified a lineage specific to European strains; African strains were scattered in various branches. Bayesian analysis allowed dating the most recent common ancestor for MmmSC around 1700. The strains circulating in Sub-Saharan Africa today, however, were shown to descend from a strain that existed around 1810. MmmSC emerged recently, about 300 years ago, and was most probably exported from Europe to other continents, including Africa, during the 19^th^ century. Its diversity is now greater in Africa, where CBPP is enzootic, than in Europe, where outbreaks occurred sporadically until 1999 and where CBPP may now be considered eradicated unless MmmSC remains undetected.

## Introduction

Epizootic diseases have threatened cattle since their domestication. Two of them played a prominent role in the last three centuries: rinderpest and contagious bovine pleuropneumonia (CBPP), threatening the livelihood of whole populations [1] and hindering international cattle trade. However, while rinderpest has been successfully eradicated world-wide [2], CBPP is still present in many African countries.

Although the spread of CBPP in Europe and throughout the world in the middle of the 19^th^ century is well documented, little is known about its origin [3]. The earliest documents by “Testienne” and Scheuchzer do not contain precise descriptions that could be unequivocally linked to CBPP [4], [5]. The first unambiguous description of the disease may well be that of B de Haller in Switzerland in 1773 [6]. In any case, historical documents on CBPP do not provide any indication regarding the date of appearance of the disease or whether it existed from time immemorial.

Similar questions can be raised regarding the origin of CBPP in Africa. Its introduction in the southern part of the continent in 1853 is well documented [3] but there is no information regarding the existence of the disease prior to colonial times. Fulani herdsmen in Senegal had developed an original inoculation procedure to combat CBPP, by inserting pieces of infected tissue under the skin of the bridge of the nose. The resulting inflammation often caused the formation of “third horns”, which led to the description of a “new” bovine race (“*Bos triceros*”) by French zoologists [7]. This site of inoculation was unknown in Europe, where inoculation procedures were performed at the tip of the tail [8] Hence, de Rochebrune (1885) suggested that local tribes had known CBPP before colonial times [9].

In many countries in Sub-Saharan Africa, CBPP control is based on vaccination alone, but this strategy does not eradicate the disease. In fact, CBPP has recently re-entered countries, such as Tanzania, that had earlier been considered disease-free. Northern Africa has always been CBPP-free and countries in Southern Africa, such as Botswana, maintain their CBPP-free status by strict stamping-out policies. In the other continents, such as Northern America and Europe, CBPP was successfully eradicated between 1896 and 1935 by strict stamping-out programs; Australia initiated control by vaccination before adopting stamping-out policies. In Southern Europe the disease persisted in the Iberic peninsula, with outbreaks occurring sporadically at 10–15 years of interval. The reoccurrence of CBPP in Europe in the early 1990s was quite unexpected, especially for Italy where the risk of contamination should have been minimal because it borders exclusively CBPP-free countries. Classical epidemiology studies failed to explain the origin of these outbreaks [10]. At that time there was a lack of genetic tools to detect, identify, and subtype precisely *Mycoplasma mycoides* subsp. *mycoides* “Small Colony” (MmmSC), the causative agent of CBPP.

Recent genetic data may bring new insights into epidemiological questions. Molecular typing has been instrumental in determining the population structure and evolution of bacterial pathogens. Multi-locus sequence typing (MLST) [11] was first developed as a typing tool. But, being based on sequence data from housekeeping genes that are not prone to horizontal gene transfer (HGT), MLST can be used to reconstruct phylogenies. The relevance of this technique depends on the mutation rates within the selected genes and on a long-enough evolutionary period to afford sufficient variability. However, highly pathogenic bacteria are often genetically monomorphic and their lack of diversity makes it difficult to investigate their evolutionary histories [12]. MmmSC is one of the five species and subspecies composing the so-called “mycoides” cluster [13]. Taxonomic changes followed a thorough phylogenetic analysis of this cluster based on five housekeeping gene sequences [14]. Before that study, MmmSC was considered just a biotype of *M. mycoides* subsp. *mycoides*, together with the “Large Colony” biotype. The latter biotype was thereafter united to the much more variable *M. mycoides* subsp. *capri* (Mmc), leaving the “Small Colony” strains as sole representatives of *M. mycoides* subsp. *mycoides*. MmmSC constitutes an extremely monomorphic clade within the species *Mycoplasma mycoides* and the MLST analysis was not sufficiently discriminating to distinguish MmmSC strains.

MmmSC subtyping was achieved by other techniques such as multi-locus sequence analysis, which is based on alternative, more variable molecular markers selected after comparison of two whole genome MmmSC sequences [15], and multi-locus variant analysis, which is based on variable numbers of tandem repeats [16]. However, these two techniques are not suited for phylogenetic inference because they target DNA sequences without a constant molecular clock, in which sites may present very high mutation rates, possibly leading to homoplasies [17] [18].

Large-scale genomic approaches now allow the reconstruction of phylogenies for bacterial pathogens with very low mutation rates such as *Yersinia pestis* [19] and *Salmonella typhi* [20] or for taxa that have evolved recently and therefore did not accumulate high amounts of mutations [21]. In this work, next-generation sequencing technologies were applied to perform global comparisons of MmmSC strains representing, with the limitation of historical strain availability, the overall diversity of this monomorphic pathogen. The objectives of this study were to construct a robust phylogeny for this pathogen, establish when the most recent common ancestor of all MmmSC strains appeared, determine whether CBPP existed in Africa or, more likely, it was imported from Europe during colonization, and find the origins of the CBPP outbreaks that occurred in Southern Europe in the last decades.

## Results and Discussion

### Phylogenetically informative set of genes from the MmmSC core genome

The genome of six MmmSC strains from Africa and Europe was sequenced by next-generation sequencing technologies and assembled into drafts comprising 34 to 76 contigs. After annotation, the MmmSC core genome comprised 473 genes with a predicted function, 249 of which showed identical amino acid sequences and thus were not further included in the study. From the 224 remaining genes, 162 were also removed, notably because of complete or partial intragenomic duplications that may have biased SNP search. Sixty two genes were finally retained (Table S1). These results show the very low diversity among MmmSC genomes. Concatenation of the 62 genes resulted in an 83,601 bp-long sequence for all strains studied except for PG1^T^, which had an additional codon within the gene *phnE*. The concatenated sequence represented about 1/15 of the total genome length.

**Figure 1:**
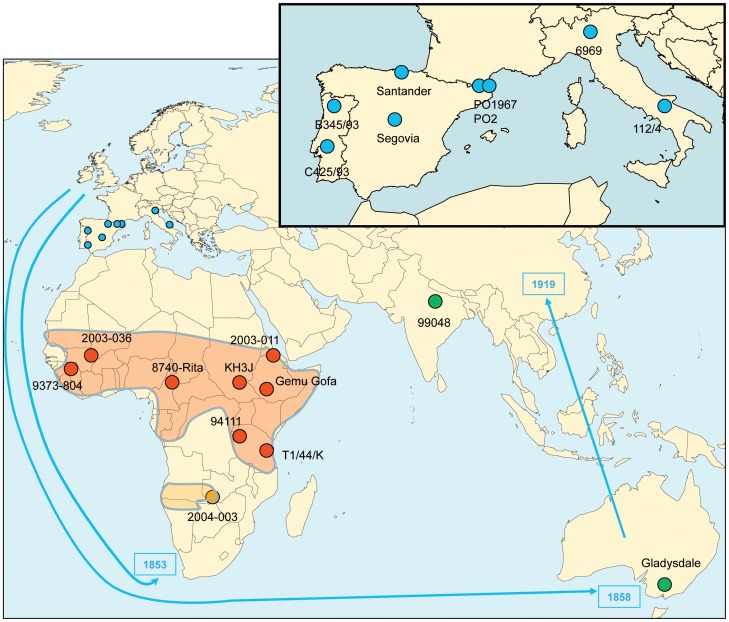
Geographical distribution and diversity of the *M. mycoides* subsp. *mycoides* “Small Colony” (MmmSC) strains studied. Twenty MmmSC strains were included in this study. Two strains were chosen from each European country that was infected in the 20th century (blue dots). Eight strains from Sub-Saharan Africa were selected to reflect the widest geographical diversity in this region (red dots). One strain was chosen to represent the southern part of the African continent (orange dot). In addition, one strain from Australia and one from India were also included (green dots). The reference strain, PG1^T^, is not represented as its origin is not known. The arrows indicate the routes of contagious bovine pleuropneumonia (CBPP) expansion during the 19th century as inferred from historical documents. The dates of introduction are indicated at the tip of the arrows. The red and orange shaded areas in Africa represent the most probable zones where CBPP is now enzootic.

Additional MmmSC concatenated sequences were obtained from 14 strains representing the diversity of this pathogen worldwide (Figure 1, Table S2). Similar numbers of strains were obtained from Europe and Africa and, for this continent, strains originated from all the sub-regions where CBPP is present. Alignment of the concatenated sequences of the final 20 MmmSC strains resulted in the identification of 139 SNPs, which were the basis for phylogenetic and molecular dating analyses. Most of these SNPs were non synonymous (N = 122), essentially due to our initial gene selection process. Besides, there was no indel nor any additional stop codon leading to truncation of predicted proteins. Strain GM12, belonging to subspecies Mmc, the closest relative of MmmSC, was used as outgroup for phylogenetic analysis. Addition of this sequence to the alignment procedure resulted in a dramatic increase in the number of SNPs (2,716) although the similarity between GM12 concatenated sequence and that of MmmSC averaged 97%.

Accurate phylogenetic inference relies on the fact that detected SNPs result from mutation and not from recombination, which may bias the phylogenetic inference and the molecular dating process. As Enright and Spratt said, “Bacteria have quite variable sex lives, ranging from near celibacy to evident promiscuity” [22]. In the case of ruminant mycoplasmas, horizontal gene transfer (HGT) had already been evidenced between *M. agalactiae* and the “mycoides cluster”, whose sole available genome was that of MmmSC at that time [23]. This HGT may probably have occurred between *M. agalactiae* and Mmc, which colonize the same hosts and organs. By comparison, recombination between MmmSC strains is very unlikely, since the genes *recG* and *recR* are truncated in MmmSC genomes, which may preclude homologous recombination [24]. Furthermore, the coexistence of two or more MmmSC strains within a single animal is very improbable. CBPP transmission can take place only between infected and naïve animals, whereas those that recover are completely immune which would hamper the multiplication of any MmmSC strain. In addition, recombination between MmmSC and other bacterial species, including the closest relative Mmc, should have been easily spotted, as it would have led to an accumulation of SNPs in the implicated genes. However, clusters of SNPs were not observed in this study. The selected gene-set was therefore suitable for phylogenetic inference.

### Phylogeny of *Mycoplasma mycoides* subsp. *mycoides* “Small Colony”

**Figure 2:**
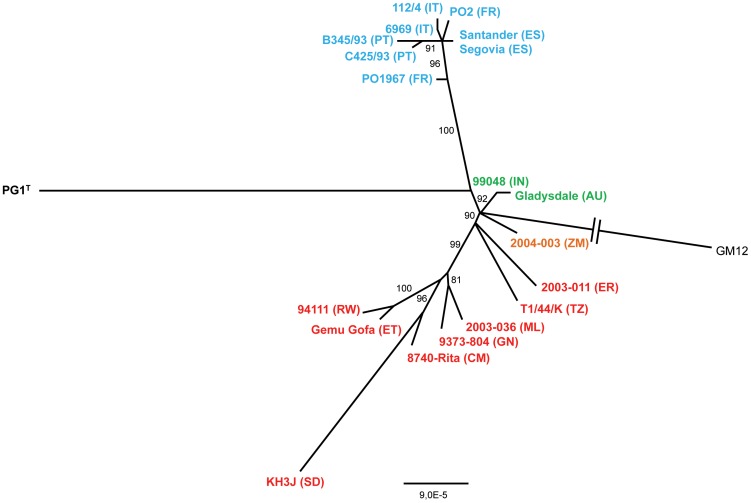
Phylogeny of *Mycoplasma mycoides* subsp. *mycoides* “Small Colony” (MmmSC) inferred by the maximum likelihood method. The maximum likelihood tree was reconstructed using PhyML (GTR with invariable sites) based on the alignment of 62 concatenated core genes of MmmSC. Bootstrap values >80% are shown. Strain names are colored according to the sampling location (see Figure 1). Country codes are indicated in brackets. The branch corresponding to the outgroup (GM12) was shortened, as indicated by two parallel bars. The scale indicates the number of substitutions per site. Abbreviations: AU = Australia; CM = Cameroon; ES = Spain; ER = Eritrea; ET = Ethiopia; FR = France; GN = Guinea; IN = India; IT = Italy; ML = Mali; PT = Portugal; RW = Rwanda; SD = Sudan; TZ = Tanzania; ZM = Zambia.

A phylogenetic tree was inferred from the concatenated nucleotidic sequences of the 20 selected MmmSC strains and an Mmc outgroup by using PhyML (Figure 2). The topology of this tree was confirmed by other methods such as maximum parsimony using Dnapars (Figure S1) and statistical parsimony using TCS (Figure S2). Yet, congruence of the phylogenies obtained by different methods is not always achieved [25]. In the case of MmmSC, the congruence could be explained by a very limited degree of genetic variation, which makes homoplasy highly improbable in our dataset. All the European strains were grouped within a single long branch supported by a high bootstrap value and may be considered a lineage. Interestingly, all strains isolated after 1980 derived from a common ancestor. This finding showed that a single strain may have spread in Southern Europe (France, Spain, Portugal and Italy) between 1980 and 1993. Further proof for this common origin was that all these strains presented the same 8.8 kb deletion [26], which must have occurred in that ancestor. In each of these European countries, the descendants from this common ancestor presented 1 to 5 additional SNPs and this could sometimes give an indication of the filiations of the strains. For example, strain 112/4 isolated in Puglia presented an additional SNP as compared to strain 6969 isolated in Lombardia and 112/4 is therefore a direct descendant from strain 6969. The most likely explanation is that Puglia was contaminated by animals from Lombardia, where CBPP was first detected in Italy and where the disease may have been present since 1988 [10]. Within our subset of MmmSC strains, we did not identify any bearing the “recent” European ancestral sequence and the 8,8 kb deletion. This would have given the origin of the CBPP outbreaks in Europe after 1980. It is unlikely the origin of these outbreaks can be found, given the paucity of MmmSC strains in laboratory collections, especially those from 1967–1980, when CBPP was not reported. CBPP control in Europe has been based on detection and stamping-out of affected herds. In this context, ancestral genotypes certainly did not persist over time, resulting in a characteristic tree topology presenting strains clustered at the tip of a long branch, which is consistent with a “bottleneck and broomstick” kind of evolution [27]. In the case of CBPP in Europe, the long-term unnoticed persistence of some strains may be related to their lower virulence, survival in an unusual host such as small ruminants, or the use of antibiotics that may mask the infection.

Five branches were located at the vicinity of the root of the tree. This was the case for two branches corresponding to strains from Australasia (Gladysdale and 99048) and from Zambia (2004-003), which are known to have been imported from Europe in the 19^th^ century [3]. These strains are then the direct descendants of the strains that prevailed in Europe at that time. The close proximity of the genotypes found in Australia and India also agrees with the introduction of CBPP in Asia with milking cattle exported from Australia. The disease entered India in 1910, China in 1919, and from Mongolia, it reached Japan in 1924 [28]. These countries are now considered CBPP-free. Two other branches near the root of the tree corresponded to strains isolated in East Africa, namely Eritrea (2003-11) and Tanzania (T1-44). Their ancestor may have been very similar to the ancestral genotype found in Europe when CBPP was exported to Australia. The fifth branch near the root corresponded to type strain PG1^T^ and was particularly long, but this may reflect extensive in-vitro passages rather than natural evolution. Finally, another highly supported branch grouped six strains isolated from Sub-Saharan Africa. These strains originated from West Africa (Senegal and Mali), Central Africa (Cameroon and Southern Sudan), and East Africa (Rwanda and Ethiopia) (Table S2), indicating that their common ancestor may have gradually invaded the African Continent. In contrast to Europe, CBPP control in Sub-Saharan Africa is based on vaccination. But vaccination is insufficient to effectively control the disease, hence allowing diverse genotypes to persist over time. This may explain why the overall diversity of African strains is greater than that found among recent European strains. That East African strains are distributed in separate, dissociated groups may be correlated with several introductions in that sub-region.

In spite of the very low genetic diversity within MmmSC strains, many branches of the trees that were generated in our study were supported by high bootstrap values. This is due to the concatenation of a high number of genes, 62, as opposed to classical MLST approaches that include only 7 to 8 gene fragments. The use of next-generation sequencing technology was central to this phylogenetic approach but it also paves the way for the rapid, robust and fine typing of MmmSC strains.

### MmmSC divergence time estimations

**Figure 3:**
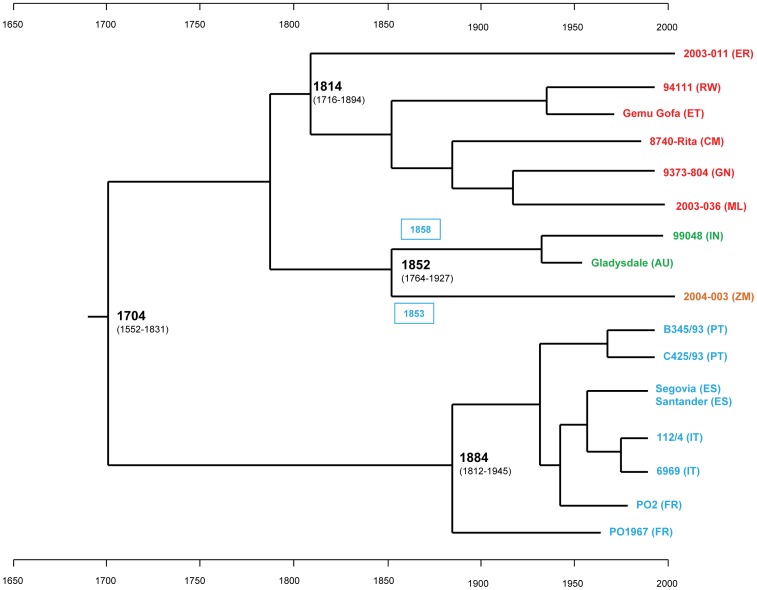
Bayesian inference of *Mycoplasma mycoides* subsp. *mycoides* “Small Colony” evolutionary history. The maximum credibility tree resulted from BEAST analysis with the concatenated sequence of 62 core genes and using a strict molecular clock and a GTR+I substitution model. Branches are scaled by time according to the scales displayed at the bottom and top of the figure. Strain names are colored according to the sampling location (see Figure 1). Country codes are indicated in brackets (for abbreviations refer to Figure 2). The dates at the nodes refer to the most recent common ancestor (MRCA) estimated by BEAST for all strains deriving from these nodes. The 95% Highest Posterior Densities (HPD) values are displayed in brackets. The node leading to the Australasian and South African strains was used as a control of estimation accuracy. The model dates their MRCA around 1850. This agrees with the dates of CBPP introduction in Australia (1858) and Southern Africa (1853), indicated in blue boxes. According to this model, the MRCA for all the tested strains emerged around 1700 and the MRCA for all the Sub-Saharan strains around 1810.

The divergence time for the MmmSC strains was estimated using BEAST, which performs a Bayesian Markov chain Monte Carlo (MCMC) analysis and infers time-measured phylogenies through molecular clock models. The initial tests used a strict molecular clock, a GTR+I substitution model as established by Modeltest and a mutation rate of 5 10^−7^/site/year as a prior. BEAST generated a phylogenetic tree and a date estimate for each of the nodes of the tree (Figure 3) taking into consideration the isolation dates of the strains. The overall tree topology was very similar to that of the tree generated by PhyML except that BEAST is not able to generate multifurcations. This slightly altered the branch topology of European strains but did not prevent the use of BEAST-generated trees to date ancestral nodes.

The date estimate of the node leading to the South African and Australasian strains was used to evaluate the model accuracy. BEAST-estimated mean time for the most recent common ancestor (tMRCA) for these strains was 1852. This agrees well with historical data establishing that CBPP was imported in those continents exactly in 1853 and 1858 [3] and that the MRCA must have emerged shortly before. The range for the 95% upper and lower highest posterior densities (95% HPD), containing 95% of the sampled values generated by BEAST, expanded from 1764 to 1927, and the mean value, 1852, was in complete agreement with historical data.

The estimated date of emergence of the MRCA of all MmmSC strains was 1704. The 95% HPD range was established at 1552 to 1831 These values are also in agreement with historical data as CBPP was already identified in 1773 by B de Haller [6], which makes the 1704 estimate very plausible. Furthermore, the lowest value of the 95% HPD range (1552) clearly rules out the possibility that CBPP may have been evolving in Africa since cattle domestication and introduction into that continent before colonial times. In Africa, the first wave of bovine introduction from the Middle-East occurred around 4000 years BC for *Bos taurus* and the second between 2000 years BC and 700 years AD for *Bos indicus* [29].. A recent study indicated that this could have been the case for the ancestor of the “mycoides cluster” [30].

The estimated date for the emergence of the MRCA of all African strains, except those exported to South Africa, was 1814 (95% HPD: 1716–1894). At this time, CBPP was well known in Europe and started to expand over the continent. Notably, CBPP affected milking cattle in the Paris region [31]. The expansion of CBPP from Europe to other continents or islands is well documented [32]: Great Britain in 1841, USA in 1858, Australia in 1858. Contrarily to what was suggested in 1885 after the discovery of “*Bos triceros*”, dating by BEAST strongly suggests that CBPP was not indigenous to Africa “from time immemorial” and that it was most likely introduced from Europe, as for the other continents. The first European colonial settlements were established as early as 1462 in the Islands of Cape Verde, followed by numerous settlements in West Africa during the 16^th^ century. European settlers may have imported cattle at some time for milk production. CBPP may therefore have been introduced in Africa any time between the 16th and 19th centuries, and BEAST analysis suggests probably around 1814.

The date of emergence of the MRCA of all European strains, 1884, is very recent considering that CBPP was present in Europe at least since 1773. The stamping-out strategies certainly wiped out many MmmSC ancestral strains, thus drastically reducing the variability within MmmSC strains in Europe.

The robustness of the molecular dating analysis by BEAST was evaluated by performing multiple trials using various molecular clock priors (10^−9^ to 10^−6^/site/year) and substitution models (HKY, GTR, NT93). Modifying these settings did not significantly alter the final estimated mutation rate for this set of genes (posterior probability, 5 10^−7^/site/year) or the dating evaluation for the tMRCA. However the 95% HPD range was strongly influenced by priors on the type of variation around the mean mutation clock rate. In bacteria, mutation rate has been estimated for decades *in vitro* by detecting phenotypic traits that depend on SNPs, such as antibiotic resistance. The *in vitro* mutation rate for mycoplasmas is similar to that of other bacteria [33]. This finding is surprising since mycoplasmas lack the methylation-mediated DNA repair system commonly found in other bacteria whose genome has not been drastically reduced in size. Mycoplasmas must have found alternative ways to correct errors occurring during DNA replication, putative candidates have recently been identified [34]. Estimates of *in vitro* mutation rates certainly do not reflect what happens *in vivo*. However, the new-generation sequencing techniques offer ways to evaluate this parameter, provided multiple strains or isolates are obtained sequentially. Quite surprisingly, *M. tuberculosis* seems to have similar mutation rates during latency periods compared to active disease [35]. The increased rate of mutation during phases of slower replication could be linked to reduced metabolism and DNA repair. Mutation rates estimated in multiresistant *Staphylococcus aureus* are in the range of 10^−5.5^/site/year [36], 10^−6^/site/year for housekeeping genes of *Helicobacter pylori* [37] and those of *Yersinia pestis* in the range of 2.3 10^−8^ to 2.9 10^−9^/site/year [19]. BEAST outputs indicated that for MmmSC and our set of genes, the most probable rate is 5 10^−7^/site/year.

### Conclusion

The CBPP agent was estimated by BEAST to have emerged around 1700 AD. This is a very recent evolutionary event and historical data indicate that it took place most probably in Europe. During colonization, CBPP was exported from Europe to all other continents, with the exception of South America, and today it remains enzootic in many African countries, where eradication has not been feasible. This emergence may have resulted from adaptation of the MmmSC ancestor, presumably a small ruminant pathogen showing a wide tropism like Mmc to a new host, cattle, and to a specific organ, the lung. This hypothesis was sustained by MmmSC and Mmc whole genome comparisons showing large DNA fragment duplications, presence of numerous insertion sequence copies, and extensive gene decay within the MmmSC genome as compared to Mmc [24]. These events are classically associated with a recent adaptation to a new host. The fate of MmmSC evolution now clearly depends on the effectiveness of CBPP control strategies. The European lineage described in this study may already be extinct if European stamping-out policies were successful. If some MmmSC strains persist unnoticed, as they did previously, new CBPP outbreaks may occur in the future. By contrast, CBPP is now expanding in Africa due to insufficient control, and MmmSC strains are likely to evolve in that continent for some time until effective measures are implemented.

## Materials and Methods

### Sampling of strains

A total of 20 MmmSC strains were used (Table S2), including 8 European and 9 African strains representing the distribution of CBPP in these continents (Figure 1). Two European strains were selected from each of the countries that declared CBPP between 1967 and 1993. African strains from each of the regions where CBPP is enzootic and representing different MLSA sequence types [15] were selected to maximize the genetic diversity. Two strains from Australasia and type strain PG1^T^ were also included. Field strains isolated at CIRAD were cloned thrice to ensure purity and subcultured less than 5 times before total DNA extraction and genomic sequencing.

### Selection of gene targets

Six MmmSC strains were initially used to select a phylogenetically informative set of genes. The genome of three strains of African origin (Gemu Gofa, 8740, and KH3J) and three strains of European origin (PO1967, B345/93, and C425/93) were sequenced in collaboration with Genoscope (Centre National de Séquençage, Evry, France), using both 454 mate-pair (Roche) and Solexa (Illumina) technologies. Sequence assembly was performed using Newbler (2.3). The resulting scaffolds were then annotated into a customized version of the CAAT-Box annotation platform [38], using the published sequence of PG1^T^ (NC_005364) as reference and following the annotation process previously described [24]. To do so, the sequence of strain PG1^T^, which had been annotated in 2004, was re-annotated beforehand using CAAT-Box and incorporated into the gene selection process.

From the core genome of these seven MmmSC strains, 62 genes were selected that had a predicted function and showed some degree of polymorphism in their amino acid sequence (Table S1). Pseudogenes and duplicated genes were excluded, as were genes coding for membrane proteins or restriction enzymes, and those prone to horizontal transfer [23]. Nucleotide sequences of the selected genes were then concatenated following the reading frame, resulting in an 83,601 bp sequence. In the case of consecutive genes with overlapping coding sequences, the upstream gene was truncated at the C-terminus end.

### Data set collection

The genomes of twelve additional MmmSC strains were sequenced in single reads on an Illumina HiSeq 2000 (GATC, Constanz, Germany). Reads from the 62 selected genes were then extracted from raw data by mapping on a reference sequence using Seqman NGen (2.0) software (DNASTAR, Madison, WI). This reference consisted of the concatenated, annotated sequence of strain PG1^T^ including flanking regions for each of the selected genes (122722 bp) to allow the correct mapping of illumina sequences on the whole gene length. On average, a read depth of 500× was obtained for each genome. This procedure allowed visual verification of the sequencing depth and of incongruities on all coding sequences by using Seqman (Lasergene 8) software (DNASTAR). Non-coding flanking regions were trimmed according to gene annotations of the consensus sequence (Seqbuilder, Lasergene V8.1.2). Additionally, selected genes were extracted from the published genome sequences of strains PG1^T^ (NC_005364) and Gladysdale (CP002107) and concatenated as described previously. Finally, GM12, a strain of the subspecies Mmc, the closest relative of MmmSC, was chosen as outgroup and corresponding sequence data were retrieved from Genebank (CP001668).

### Phylogenetic analyses

The concatenated sequences of 62 genes were aligned using ClustalW as implemented in Seaview V4.3.2 [39], resulting in a supermatrix (Figure S3). Three tree-building methods were used to reconstruct the phylogeny of MmmSC from this supermatrix: maximum likelihood (ML), maximum parsimony (MP), and statistical parsimony. ML analyses were performed with PhyML V3.0 [40] available on the web (http://www.atgc-montpellier.fr/phyml/). The general time-reversible model including estimation of invariant sites (GTR+I) was applied, since it was identified as the best-fit substitution model by Modeltest V3.7 [41]. Node support was assessed with the bootstrap technique using 100 replicates. An unrooted parsimony tree was inferred using Dnapars from Phylip package V3.69 (http://evolution.genetics.washington.edu/phylip.html) [42] based on alignment of SNPs only (Figure S4). One thousand bootstrap replicates were created by Seqboot (Phylip) and analyzed by Dnapars for multiple dataset. The consensus tree was built by Consense (Phylip). A statistical parsimony network was estimated using TCS V1.21 [43] based on the alignment of polymorphic sites only with a fixed connection limit set at 20.

### Divergence time estimates by Bayesian evolutionary analysis

BEAST V1.6.1 (http://beast.bio.ed.ac.uk) [44] was used to estimate the divergence time of the various clades. Sequences from strains that had been subcultured an unknown number of times *in vitro* (type strain PG1^T^ and vaccine strains T1/44/K and KH3J) were removed from the alignment, since they may have evolved with a different molecular clock as compared to field strains. Owing to the low mutation rate in MmmSC strains, we assumed that small number of *in-vitro* passages needed for isolation and identification of field strains would not bias *in-vivo* molecular clock and time divergence estimates. Samples were dated according to their year of isolation when it was known. Strain Gladysdale was dated around 1956 according to historical data showing that the state of Victoria had been freed from CBPP at an earlier stage than the Northern Australian states [45]. The substitution model used was similar to that of the PhyML analysis, GTR+I, using a strict molecular clock. The mean clock rate was set at 5 10^−7^, as determined by initial runs of BEAST,.and variation around this mean followed a normal distribution. An exponential-growth model was used as a prior for tree building. The MCMC chain was set at 70 million generations with auto-optimization and sampled every 1000 generations. The input file showing all parameters for divergence time estimation was generated using BEAUTi (BEAST) (Figure S5). After appropriate burn-in cutoff (N = 7000), the effective sampling size was estimated by the graphical application Tracer (BEAST package) (Effective Sample Size>200). A single target tree was produced by TreeAnnotator (BEAST package) and analyzed by Figtree V1.3.1 (http://tree.bio.ed.ac.uk/) to produce a chronogram. Mean Node Heights were recorded, as were 95% Highest Posterior Densities (HPD).

## Supporting Information

Figure S1 **Phylogenetic tree of** ***Mycoplasma mycoides*** **subsp.** ***mycoides*** **“Small Colony” (MmmSC) sequences using parsimony analysis.** The most parsimonious tree was obtained using Dnapars (Phylip package) from the alignment of the 139 nucleotides corresponding to the MmmSC polymorphic sites of 62 concatenated core genes. Strain names are colored according to the sampling location (see Figure 1). Country codes are indicated in brackets. The branch corresponding to the reference strain, PG1^T^, was shortened. Bootstrap values over 75% are indicated. The probable ancestral nodes, located within the circled region “A”, were inferred from their position at the center of gravity of the tree. All strains of European origin were found within a single lineage. Furthermore, the most recent European strains are on branches at a multifurcation, at the tip of the lineage, with a single ancestor, circled “B”. This is evidence of the clonal expansion of this ancestor giving rise to the recent isolates. By contrast, strains from Sub-Saharan Africa are at the extremity of bifurcating branches, an indication of the greater variability in that continent. The South African or Australasian strains are on short branches originating from the probable ancestral nodes. Abbreviations: AU = Australia; CM = Cameroon; ES = Spain; ER = Eritrea; ET = Ethiopia; FR = France; GN = Guinea; IN = India; IT = Italy; ML = Mali; PT = Portugal; RW = Rwanda; SD = Sudan; TZ = Tanzania; ZM = Zambia.
(TIF)

Figure S2 **Haplotype network of** ***Mycoplasma mycoides*** **subsp.** ***mycoides*** **“Small Colony” (MmmSC) sequences obtained using statistical parsimony.** The cladogram was obtained with TCS from the alignment of the 139 nucleotides corresponding to the MmmSC polymorphic sites of 62 concatenated core genes. Strains KH3J and PG1^T^ were not connected to this network because the connecting distance was limited to 20 steps. Each segment corresponds to one mutational step. The color of the strain names refers to their geographical origin (see Figure 1). Country codes are indicated in brackets. The probable root of the network, circled “A”, was predicted by the number of connections and the position within the network. The two possible roots here have 4 connections. They are hypothetical haplotypes, while most of the strain haplotypes are tip haplotypes. These ancestral haplotypes may not have persisted until today because of the CBPP control strategies based on stamping-out methods, especially in Europe. One haplotype, circled “B”, is connected to all recent isolates of European origin and can be considered as the ancestor of these strains. Abbreviations: AU = Australia; CM = Cameroon; ES = Spain; ER = Eritrea; ET = Ethiopia; FR = France; GN = Guinea; IN = India; IT = Italy; ML = Mali; PT = Portugal; RW = Rwanda; SD = Sudan; TZ = Tanzania; ZM = Zambia.
(TIF)

Figure S3 **Alignment of 62 concatenated core genes of 20 MmmSC strains and an Mmc outgroup.** Input file for phylogenetic analysis using PhyML.
(PHY)

Figure S4 **Alignment of polymorphic sites of 62 concatenated core genes from 20 MmmSC strains.** Multifasta file containing only polymorphic bases, used for analysis with the PHYLIP package.
(FAS)

Figure S5 **Input file for BEAST analysis.** Xml file generated by the BEAUTi software. This file contains all sequence data as well as informations needed by the BEAST software to perform a Bayesian evolutionary analysis such as site model, clock models, priors, operators and MCMC chain length.
(XML)

Table S1 **List of MmmSC concatenated genes.**
(TIF)

Table S2 **List of** ***Mycoplasma mycoides*** **subsp.** ***mycoides*** **“Small Colony” strains studied.**
(TIF)
