# Supplementary figures and images for: Evolutionary History of Contagious Bovine Pleuropneumonia Using Next Generation Sequencing of Mycoplasma mycoides Subsp. mycoides “Small Colony”
Source: PLoS One. 2012 Oct 8;7(10):e46821. doi: 10.1371/journal.pone.0046821 (PMC3468273; doi:10.1371/journal.pone.0046821)

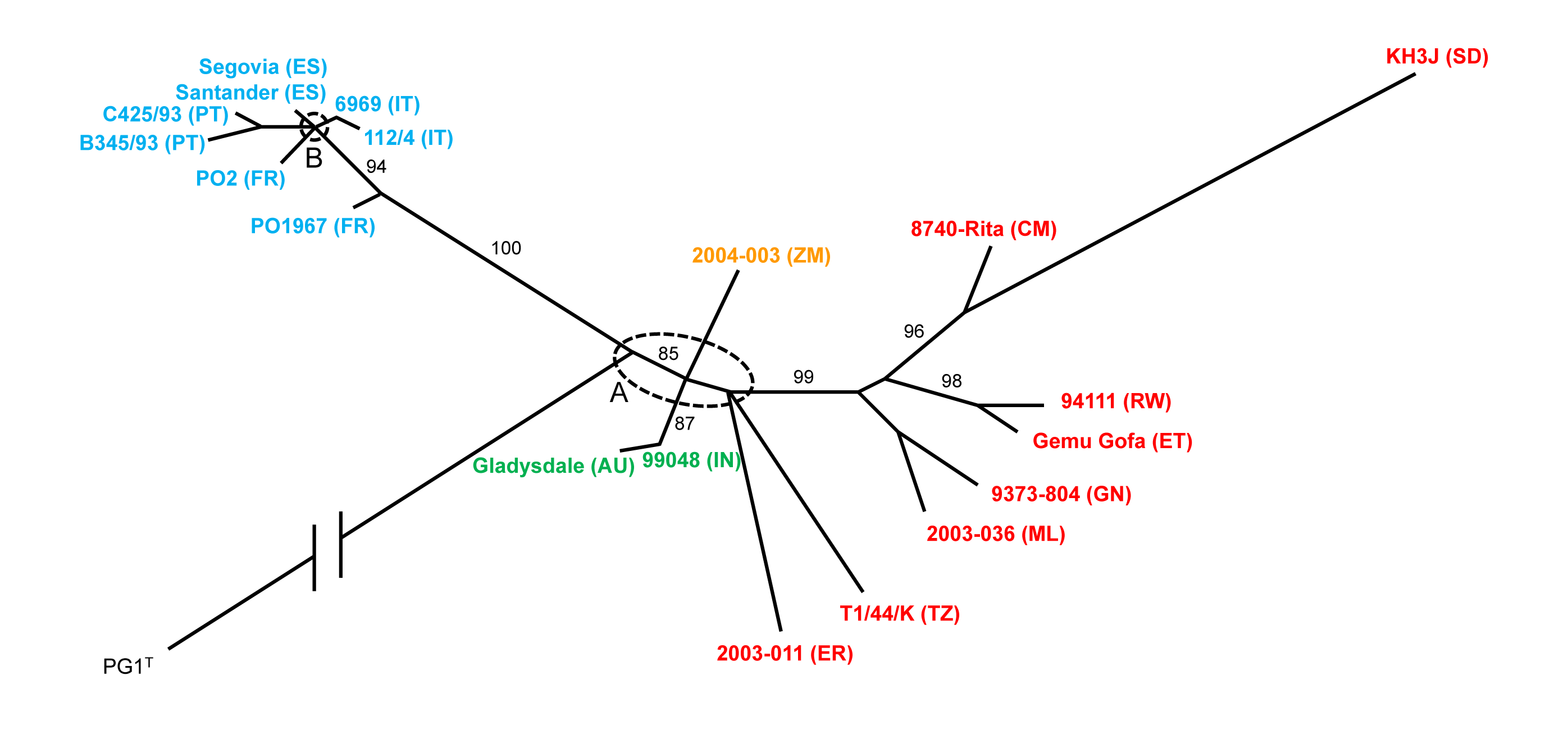

Supplement: Figure S1 — Phylogenetic tree of Mycoplasma mycoides subsp. mycoides “Small Colony” (MmmSC) sequences using parsimony analysis. The most parsimonious tree was obtained using Dnapars (Phylip package) from the alignment of the 139 nucleotides corresponding to the MmmSC polymorphic sites of 62 concatenated core genes. Strain names are colored according to the sampling location (see Figure 1). Country codes are indicated in brackets. The branch corresponding to the reference strain, PG1T, was shortened. Bootstrap values over 75% are indicated. The probable ancestral nodes, located within the circled region “A”, were inferred from their position at the center of gravity of the tree. All strains of European origin were found within a single lineage. Furthermore, the most recent European strains are on branches at a multifurcation, at the tip of the lineage, with a single ancestor, circled “B”. This is evidence of the clonal expansion of this ancestor giving rise to the recent isolates. By contrast, strains from Sub-Saharan Africa are at the extremity of bifurcating branches, an indication of the greater variability in that continent. The South African or Australasian strains are on short branches originating from the probable ancestral nodes. Abbreviations: AU = Australia; CM = Cameroon; ES = Spain; ER = Eritrea; ET = Ethiopia; FR = France; GN = Guinea; IN = India; IT = Italy; ML = Mali; PT = Portugal; RW = Rwanda; SD = Sudan; TZ = Tanzania; ZM = Zambia. (TIF) [file pone.0046821.s001.tif]

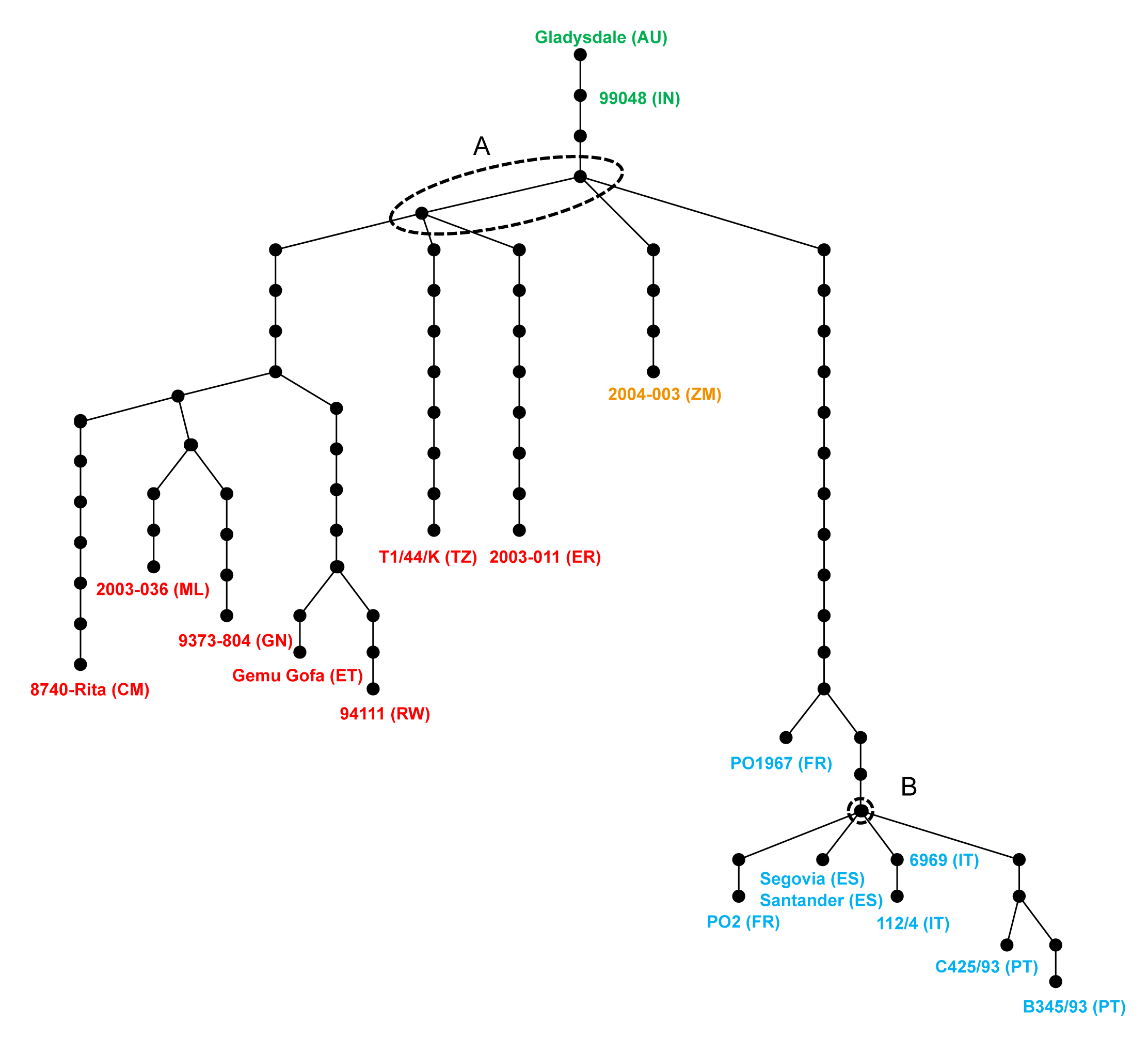

Supplement: Figure S2 — Haplotype network of Mycoplasma mycoides subsp. mycoides “Small Colony” (MmmSC) sequences obtained using statistical parsimony. The cladogram was obtained with TCS from the alignment of the 139 nucleotides corresponding to the MmmSC polymorphic sites of 62 concatenated core genes. Strains KH3J and PG1T were not connected to this network because the connecting distance was limited to 20 steps. Each segment corresponds to one mutational step. The color of the strain names refers to their geographical origin (see Figure 1). Country codes are indicated in brackets. The probable root of the network, circled “A”, was predicted by the number of connections and the position within the network. The two possible roots here have 4 connections. They are hypothetical haplotypes, while most of the strain haplotypes are tip haplotypes. These ancestral haplotypes may not have persisted until today because of the CBPP control strategies based on stamping-out methods, especially in Europe. One haplotype, circled “B”, is connected to all recent isolates of European origin and can be considered as the ancestor of these strains. Abbreviations: AU = Australia; CM = Cameroon; ES = Spain; ER = Eritrea; ET = Ethiopia; FR = France; GN = Guinea; IN = India; IT = Italy; ML = Mali; PT = Portugal; RW = Rwanda; SD = Sudan; TZ = Tanzania; ZM = Zambia. (TIF) [file pone.0046821.s002.tif]

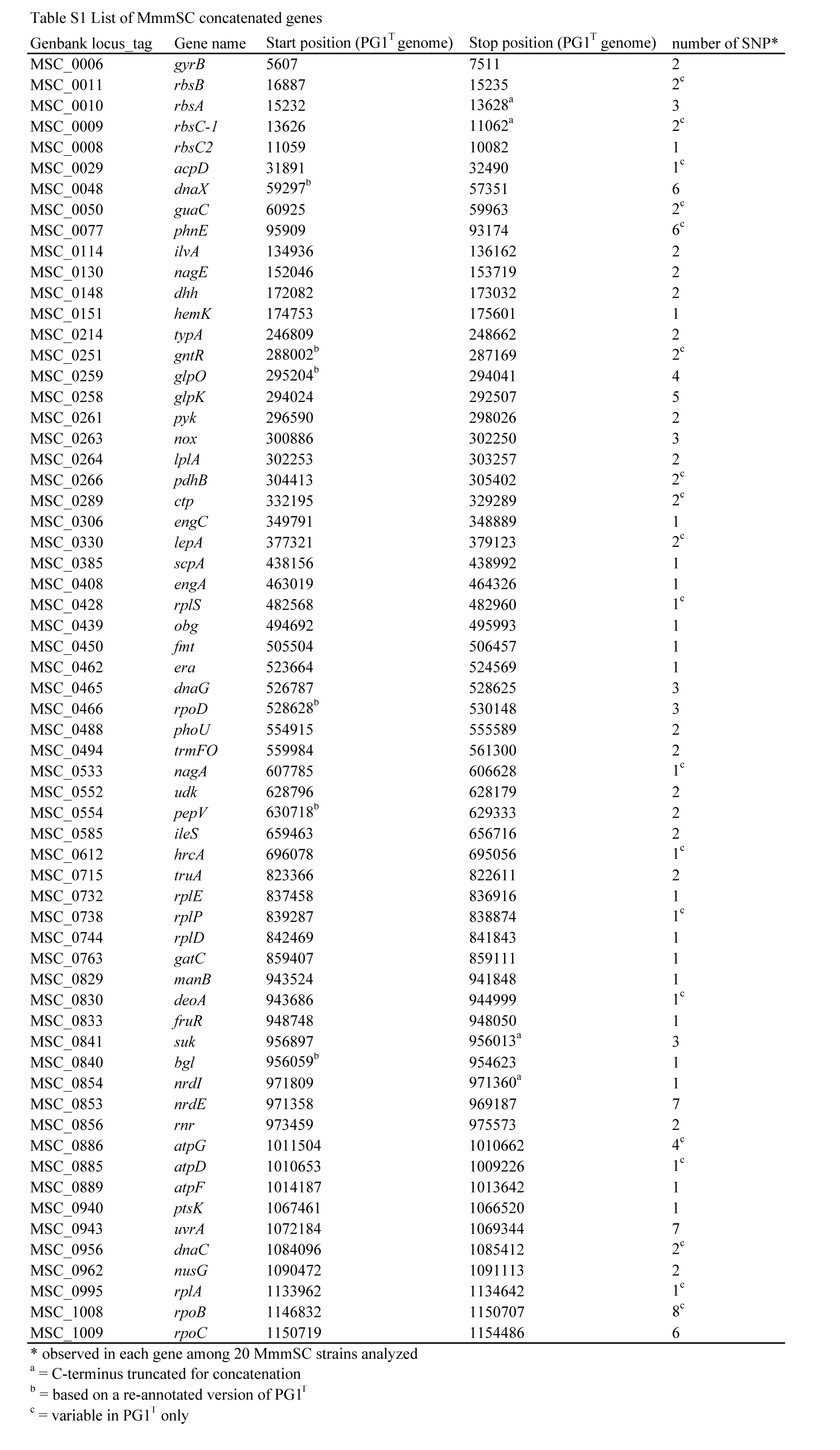

Supplement: Table S1 — List of MmmSC concatenated genes. (TIF) [file pone.0046821.s006.tif]

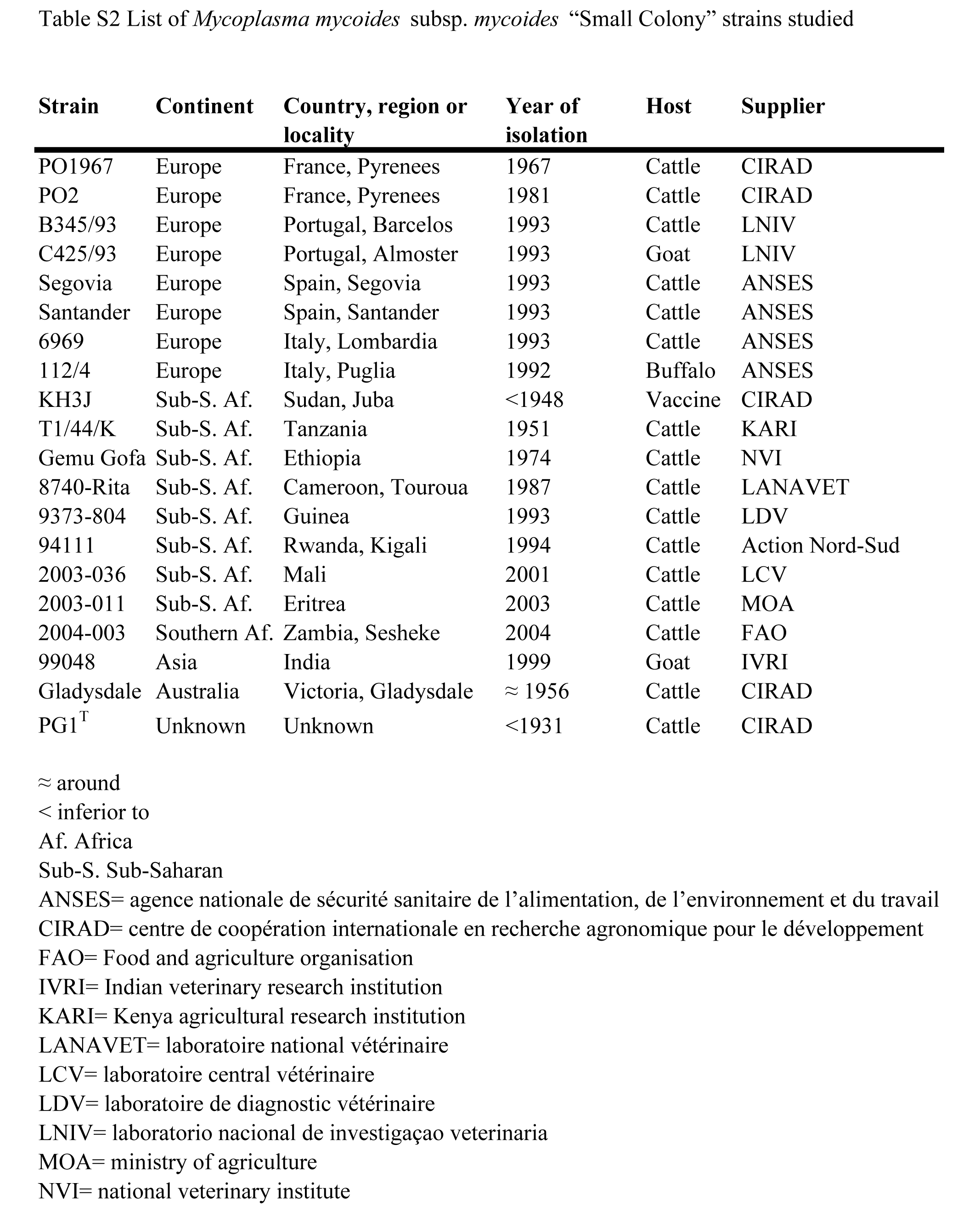

Supplement: Table S2 — List of Mycoplasma mycoides subsp. mycoides “Small Colony” strains studied. (TIF) [file pone.0046821.s007.tif]
